# Data set of activity cliffs with single-atom modification and associated X-ray structure information for medicinal and computational chemistry applications

**DOI:** 10.1016/j.dib.2020.106364

**Published:** 2020-10-01

**Authors:** Huabin Hu, Jürgen Bajorath

**Affiliations:** Department of Life Science Informatics, B-IT, LIMES Program Unit Chemical Biology and Medicinal Chemistry, Rheinische Friedrich-Wilhelms-Universität, Endenicher Allee 19c, D-53115 Bonn, Germany

**Keywords:** Bioactive compounds, High-confidence data, Activity cliffs, Single-atom modifications, Structure-activity relationships, X-ray structures

## Abstract

Activity cliffs (ACs) are defined as pairs of structurally similar or analogous active compounds with large potency differences [1]. As such, they provide important information for the exploration of structure-activity relationships (SARs) and chemical optimization. We have introduced a new category of ACs capturing minimal (single-atom) chemical modifications and identified more than 1500 of such ACs in compounds with activity against a variety of target proteins [2]. ACs with single-atom modifications (sam_ACs) include “atom-replacement ACs” (ar_ACs) that contain a single-atom replacement (N to C (N-C), O-C, N-O, or S-O) at a given position and “atom-walk ACs” (aw_ACs), in which two analogs are only distinguished by the position of a single heteroatom (non-carbon atom). For a number of sam_ACs, X-ray structures of complexes between AC targets and AC compounds were identified, which made it possible to explore the formation of sam_ACs on the basis of well-defined ligand-target interactions [2]. Our collection of sam_ACs including associated chemical and X-ray structure information, as described herein, is made freely available.

## Specifications Table

**Table:** 

Subject	Drug discovery
Specific subject area	Computational analysis of compounds, curated activity data, and X-ray structures to identify, organize, and explore new ACs
Type of data	Table (standard .csv format)
How data were acquired	Primary compound activity data were acquired from the ChEMBL database [3] and curated for the identification of sam_ACs. Crystallographic structures of AC targets in complex with AC analogs were obtained from the RCSB Protein Data Bank (PDB) [4].
Data format	Secondary data (ar_ACs, aw_ACs, and associated information) Table (consistently formatted)
Parameters for data collection	The following selection criteria were applied: (1) bioactive compounds from ChEMBL version 26, (2) *homo sapiens* as assay organism, (3) single protein as target assay type, (4) only K_i_ and IC_50_ values with “=” relationship as potency measurements, (5) valid SMILES representation [5], (6) X-ray structures of ligand-target complexes from the PDB.
Description of data collection	On the basis of data selection criteria detailed above, compounds with high-confidence activity data were selected and analog pairs with single-atom modification were enumerated. Analog pairs with an at least 100-fold difference in potency on the basis of defined potency measurement were classified as sam_ACs and organized into subset of K_i_ and IC_50_ measurement-dependent ar_ACs and aw_ACs. In addition, X-ray structures of complexes between sam_AC targets and sam_AC compounds were extracted from the PDB.
Data source location	Department of Life Science Informatics, B-IT, University of Bonn, Endenicher Allee 19c, D-53115 Bonn, Germany. Primary data sources: ChEMBL [3], PDB [4].
Data accessibility	The data set is freely available for download from the public university cloud as a formatted data file via the following link: https://uni-bonn.sciebo.de/s/ZcK1Y2vPrfNF5hC
Related research article	H. Hu, J. Bajorath, Activity Cliffs Produced by Single-Atom Modification of Active Compounds: Systematic Identification and Rationalization Based on X-ray Structures. Eur. J. Med. Chem. 2020, 207, 112846. https://doi.org/10.1016/j.ejmech.2020.112846.

## Value of the Data

• The collection of sam_ACs provides 1514 instances of pairs of nearly identical analogs that are only distinguished by a single-atom modifications, but have an at least 100-fold difference in compound potency on the basis of curated high-confidence activity data. These sam_ACs are active against a variety of therapeutically relevant targets. Hence, the newly introduced sam_ACs provide an extensive knowledge base of SAR information for medicinal chemists.
• The AC data provides a basis for specific applications. For example, for protein kinase inhibitors, G protein-coupled receptor (GPCR) ligands, or compounds with activity against other targets, sam_ACs can be inspected to identify critical atom positions and substitution sites for analog series, which can then be taken into consideration in optimization efforts on other structurally related compounds. In addition, sam_ACs are also prime candidates to aid in the identification of most important substitution sites in different compound series.
• For 59 sam_ACs, X-ray structures of complexes between AC targets and (mostly highly potent) AC analogs were identified. These structural data enable the analysis and interpretation of sam_AC formation in three dimensions. In addition, the X-ray structures make it possible to computationally probe individual ligand-target interactions involving atom modification sites in AC analogs and their energetic contributions to binding. For example, free energy perturbation methods may be applied to transform a highly potent crystallographic ar_AC compound into its weakly potent analog (or *vice versa*) and estimate the associated change in the free energy of binding. Hence, the data also provide attractive test cases for computational chemistry.

## 1 Data Description

**Fig. 1:**
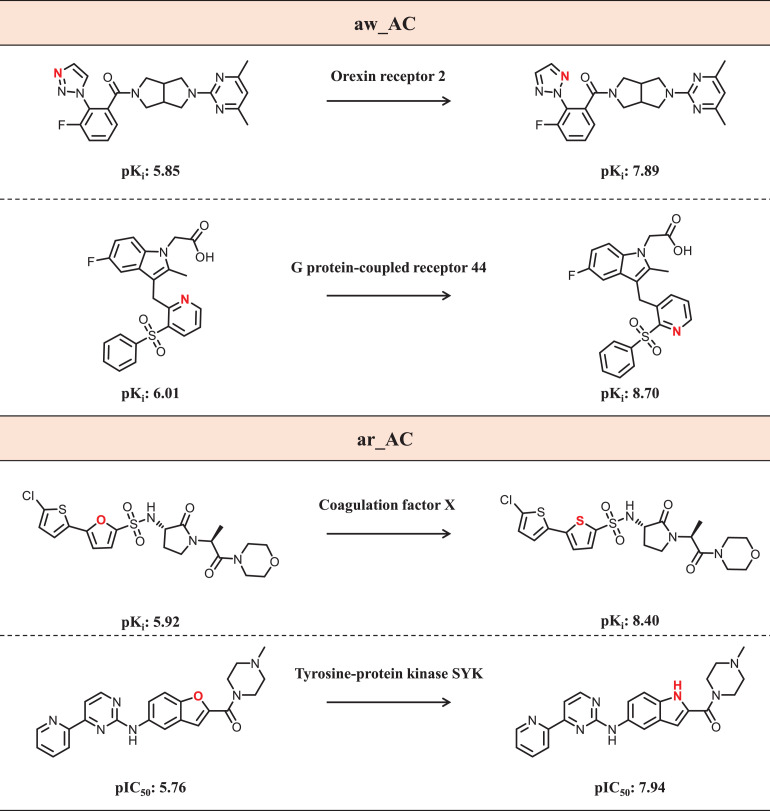
Shown are exemplary aw_ACs (top) and ar_ACs (bottom), respectively. Modified atoms are colored red. For each AC, the target and negative decadic logarithmic compound potency values (pK_i_ or pIC_50_) are reported.

The curated data set consists of a total of 1514 sam_ACs formed by 2514 unique bioactive compounds with activity against 377 targets. The target distribution of the sam_ACs is detailed in [2]. The sam_AC data set is composed of two potency measurement-dependent subsets, one of which is based on IC_50_ and the other on K_i_ data. While these types of measurements are well-defined, they cannot be directly compared and are therefore separately considered. Furthermore, sam_ACs are subdivided into ar_ACs and aw_ACs accounting for atom replacements and positional changes, respectively. Fig. 1 shows representative examples.

For 59 sam_ACs with activity against 38 targets, X-ray structures of complexes formed by AC targets and AC analogs were found. For only three of these sam_ACs, X-ray structures with both AC analogs were available. For 52 of 56 sam_ACs with only one available crystal structure, the highly potent AC analog was co-crystallized. For sam_ACs with at least one X-ray structure, class and group assignments of crystallographic targets are reported in Table 1. Most structural information was available for sam_ACs formed by kinase and protease inhibitors, with 15 and 10 instances in IC_50_ and K_i_ data set, respectively.

**Table 1:** Distribution of activity cliffs with X-ray structures across different groups of targets.

Data set	Target class	Target group	No. sam_ACs
IC_50_	Enzyme	Kinase	15
	Enzyme	Unclassified	5
	Enzyme	Protease	4
	Enzyme	Lyase	2
	Enzyme	Phosphodiesterase	2
	Membrane receptor	Family A G protein-coupled receptor	2
	Enzyme	Oxidoreductase	1
	Enzyme	Transferase	1
	Epigenetic regulator	Writer	1
	Transcription factor	Nuclear receptor	1
K_i_	Enzyme	Protease	10
	Enzyme	Lyase	4
	Epigenetic regulator	Eraser	2
	Epigenetic regulator	Reader	2
	Membrane receptor	Family A G protein-coupled receptor	2
	Enzyme	Cytochrome P450	1
	Enzyme	Isomerase	1
	Enzyme	Kinase	1
	Enzyme	Phosphodiesterase	1
	Enzyme	Transferase	1

Reported is the target group distribution of sam_ACs with at least one X-ray structure in the IC_50_- and K_i_-based data sets.

Table 2 reports the numbers of different ar_ACs and aw_ACs for which X-ray structures were available. For ar_ACs, all specified atom replacements were observed, while aw_ACs with X-ray structures were limited to nitrogen atom walk (N-C type).

**Table 2:** Atom-replacement and atom-walk activity cliffs with X-ray structures.

Type	IC_50_		K_i_	
	No. ar_ACs	No. aw_ACs	No. ar_ACs	No. aw_ACs
N-C	17	6	12	3
O-C	5	0	3	0
N-O	5	0	6	0
S-O	1	0	1	0
Total no.	28	6	22	3

Reported are the numbers of different ar_ACs and aw_ACs with X-ray structures for the IC_50_- and K_i_-based data sets.

In the formatted table (standard .csv format) of the deposited data set, the following information is provided for each of the 1514 sam_ACs:

- ChEMBL compound identifiers (IDs) of the AC analogs;
- standard unique SMILES representation [5,6] of the AC analogs;
- potency measurement type (IC_50_ or K_i_);
- negative decadic logarithmic potency values (pIC_50_ or pK_i_) of the AC analogs;
- name, UniProt [7] and ChEMBL ID of the AC target;
- target class and group according to ChEMBL target/family classification [3]
- type of sam_AC (ar_AC or aw_AC);
- type of single-atom modification (N-C, O-C, N-O or S-O);
- PBD ID(s) of complex X-ray structure with AC target/analog (if available).

## 2 Experimental Design, Materials and Methods

ChEMBL was used as the source of compounds and activity data for curation. This database, publically available and mostly originating from medicinal chemistry sources, was utilized to collect high-confidence activity data. Stringent selection criteria were applied. Only compounds forming direct interactions (assay relationship type: “D”) with human targets at the highest confidence level (assay confidence score: 9) were selected. In addition, only numerically specified equilibrium constants (K_i_ values) or IC_50_ values with exact “=” relationship were considered as potency measurements and separately organized into two data subsets. Applying these criteria, a total of 225,498 unique compounds active against 1841 targets (IC_50_-based subset) and 85,598 unique compounds active against 993 targets (K_i_-based subset) were obtained for further analysis.

Then, analog pairs with single-atom modifications (N-C, O-C, N-O and S-O) were systematically enumerated with the aid of RDKit [8]. A total of 36,526 and 17,526 target-based analog pairs were obtained for IC_50_ and K_i_ subset, respectively. Analog pairs having an at least 100-fold difference in potency were selected as sam_ACs, yielding 1006 and 508 ACs for IC_50_ and K_i_ subset, respectively, amounting to a total of 1514 newly identified sam_ACs.

Next, for each sam_AC, the ChEMBL target ID was mapped to the corresponding UniProt ID, which were then used to search for X-ray structures of complexes involving the AC target. Each pre-selected complex structure was then examined for the presence of an AC analog and qualifying X-ray structures were selected.

## Ethics Statement

This is a secondary data set and thus does not involve any human or animal testing.

## Declaration of Competing Interest

The authors declare that they have no known competing financial interests or personal relationships which have, or could be perceived to have, influenced the work reported in this article.
